# Ventilator dysfunction due to unexpected salbutamol crystallisation while using a nebuliser: a practical advice for intensivists

**DOI:** 10.1186/s40635-022-00441-y

**Published:** 2022-04-25

**Authors:** Jos L. M. L. le Noble, Frank J. M. Terpoorten, Mary-Anne Funnekotter-van der Snoek, Norbert Foudraine

**Affiliations:** 1 Department of Intensive Care, VieCuri Medical Center, Tegelseweg 210, 5912 BL Venlo, The Netherlands; 2 Department of Pharmacology and Toxicology, UM, PO Box 616, 6200 MD Maastricht, The Netherlands; 3 Department of Medical Technology, VieCuri Medical Center, PO Box 1926, 5900 BX Venlo, The Netherlands; 4 Department of Clinical Pharmacy, VieCuri Medical Center, PO Box 1926, 5900 BX Venlo, The Netherlands

**Keywords:** Ventilator malfunction, Nebuliser, Aerosol, Salbutamol, Crystallisation

## To the Editor,

In our ICU, we use a Servo-u^®^ mechanical ventilator (Getinge). During a regular pre-use check, a breathing circuit malfunction was detected along with another malfunction during the expiratory hold manoeuvre. Inspection of the ventilator outlet system revealed unexpected deposition of white crystals of unknown origin, both within the channel and on the outlet valve membrane.

A dysfunctional outlet valve membrane may disrupt accurate levels of positive end-expiratory pressure and facilitate backflow of the exhaled air, causing ventilator malfunction and harm to the patient. We hypothesised that aerosol crystallisation during pulmonary drug delivery could be a likely cause.

We performed an experimental bench study using a Servo-u^®^ mechanical ventilator connected to a test lung and investigated whether nebulisation of frequently used drugs causes crystal depositions. Standard warming and humidification were performed with an MR950 humidifier (Fisher & Paykel^®^) [1]. Two types of expiratory filters were used: standard Fisher & Paykel^®^ filters (Part No. RT019) and Getinge^®^ filters (Servo Duo Guard) [1]. Aerosol drug delivery was performed using a mesh nebuliser (Aeroneb^®^ Solo). The tested drugs were 2.0 mL ipratropium (Atrovent^®^: 500 μg/2 mL), 2.5 mL salbutamol (Ventolin^®^: 5.0 mg/2.5 mL, Nebules), 2 mL acetylcysteine (Fluimucil^®^: 100 mg/mL), and 5 ml iloprost (4 μg/mL). Saline solution (5 mL, NaCl 0.9%) served as the control. Tandem mass spectrometry (UPLC-MS/MC) and UPLC PDA-UV spectrophotometry were used to analyse the chemical composition of the crystal deposits.

**Fig. 1:**
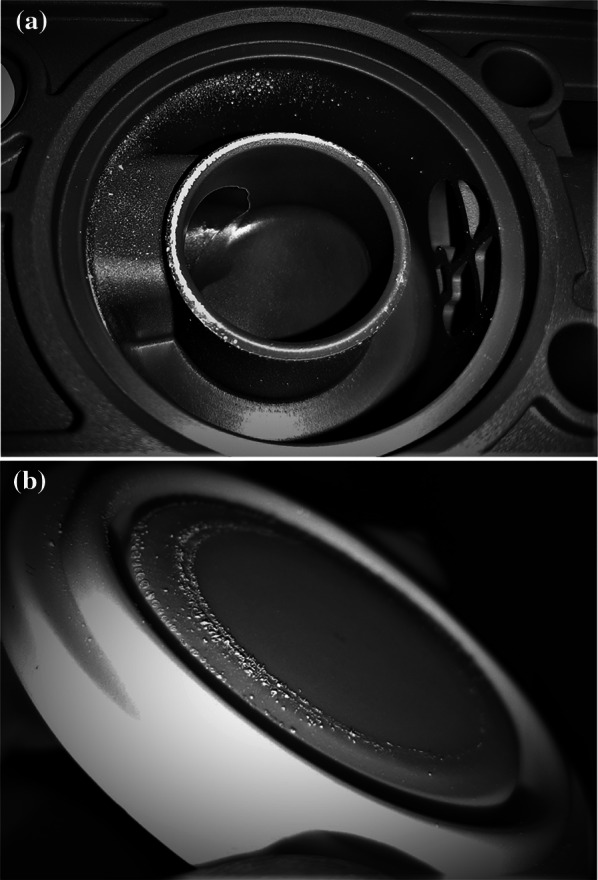
**a** Ventilator outlet system of the Servo-u^®^. Note the white deposition of salbutamol crystals which occurred following salbutamol nebulisation. The drum on top of the ventilator was removed. **b** The drum of the ventilator covered with white deposition of salbutamol crystals

Macroscopically visible crystals were observed only following Ventolin^®^ aerosol delivery using a standard Fisher & Paykel^®^ filter (Fig. 1). No deposition was observed using the Getinge^®^ filter. Further, inconsistencies in expiratory pressures were observed on the ventilator display, indicating outlet valve malfunction. Both UPLC–MS/MC and UPLC PDA-UV analyses of the crystals identified salbutamol.

The main finding of our study is that salbutamol nebulisation can lead to crystallisation in the outlet system of a ventilator (Servo-u^®^) when a Fisher & Paykel^®^ breathing circuit filter is used.

Previously, only one case report has shown intraoperative obstruction of the filter in the expiratory limb of the breathing circuit by nebulised salbutamol in paediatric patients [2].

Ventilated patients are frequently administered high doses of the short-acting β-agonist salbutamol [3, 4]. Heat and humidification may cause supersaturation and precipitation of salbutamol when cooling down in the expiratory limb [1]. Furthermore, differences in expiratory filter characteristics, a large filter pore size, and the size distribution of aerosols could account for salbutamol crystallisation [1, 5].

In our setup, the aerosol administration was not synchronised with inspiratory flow, which may have led to aerosol losses during exhalation, with large amounts of the drug being injected into the expiratory limb and engulfing the outlet valve. Therefore, a breath-synchronised mesh nebuliser has been developed which generates aerosols only during inspiration [3].

Depending on the filter used in the expiratory limb, salbutamol nebulisation may cause crystallisation in the outlet system of the breathing circuit of the ventilator. Therefore, salbutamol prescriptions in the ICU should address nebulisation practices, and aerosols should only be provided during inspiratory flow during mechanical ventilation.

## Data Availability

Not applicable.
